# Estimation of current cumulative incidence of leukaemia-free patients and current leukaemia-free survival in chronic myeloid leukaemia in the era of modern pharmacotherapy

**DOI:** 10.1186/1471-2288-11-140

**Published:** 2011-10-11

**Authors:** Tomáš Pavlík, Eva Janoušová, Zdeněk Pospíšil, Jan Mužík, Daniela Žáčková, Zdeněk Ráčil, Hana Klamová, Petr Cetkovský, Marek Trněný, Jiří Mayer, Ladislav Dušek

**Affiliations:** 1 Institute of Biostatistics and Analyses, Masaryk University, Brno, Czech Republic; 2 Department of Mathematics and Statistics, Faculty of Science, Masaryk University, Brno, Czech Republic; 3 Department of Internal Medicine, Haematology and Oncology, Faculty Hospital Brno and Masaryk University, Brno, Czech Republic; 4 Institute of Haematology and Blood Transfusion, Prague, Czech Republic

## Abstract

**Background:**

The current situation in the treatment of chronic myeloid leukaemia (CML) presents a new challenge for attempts to measure the therapeutic results, as the CML patients can experience multiple leukaemia-free periods during the course of their treatment. Traditional measures of treatment efficacy such as leukaemia-free survival and cumulative incidence are unable to cope with multiple events in time, e.g. disease remissions or progressions, and as such are inappropriate for the efficacy assessment of the recent CML treatment.

**Methods:**

Standard nonparametric statistical methods are used for estimating two principal characteristics of the current CML treatment: the probability of being alive and leukaemia-free in time after CML therapy initiation, denoted as the current cumulative incidence of leukaemia-free patients; and the probability that a patient is alive and in any leukaemia-free period in time after achieving the first leukaemia-free period on the CML treatment, denoted as the current leukaemia-free survival. The validity of the proposed methods is further documented in the data of the Czech CML patients consecutively recorded between July 2003 and July 2009 as well as in simulated data.

**Results:**

The results have shown a difference between the estimates of the current cumulative incidence function and the common cumulative incidence of leukaemia-free patients, as well as between the estimates of the current leukaemia-free survival and the common leukaemia-free survival. Regarding the currently available follow-up period, both differences have reached the maximum (12.8% and 20.8%, respectively) at 3 years after the start of follow-up, i.e. after the CML therapy initiation in the former case and after the first achievement of the disease remission in the latter.

**Conclusions:**

Two quantities for the evaluation of the efficacy of current CML therapy that may be estimated with standard nonparametric methods have been proposed in this paper. Both quantities reliably illustrate a patient's disease status in time because they account for the proportion of patients in the second and subsequent disease remissions. Moreover, the model is also applicable in the future, regardless of what the progress in the CML treatment will be and how many treatment options will be available, respectively.

## Background

Treatment guidelines and recommendations for patients treated for chronic myeloid leukaemia (CML) have changed dramatically over the last decade, as a BCR-ABL tyrosine kinase inhibitor (TKI), imatinib, was introduced in 1998 [1,2]. Since then, imatinib has been repeatedly shown to provide a higher likelihood of achieving long-term disease remissions than any other therapy [3]. Thus, imatinib has become the standard first-line treatment for chronic phase CML (CP-CML) patients, and has additionally been proven useful in more advanced phases of the disease.

However, despite its very good performance in treating CML, imatinib therapy cannot be regarded as a fully curative treatment for CML patients. Even in the era of imatinib, CML remains a chronic disease, requiring lifelong therapy with various consecutive strategies. Moreover, a probability of remaining in complete cytogenetic remission (CCyR) while still receiving imatinib 5 years after diagnosis was estimated to be approximately 63% considering intention-to-treat analysis [4]. Thus, about one third of patients may need alternative therapeutic options to imatinib, either due to resistance or intolerance. The subsequent therapeutic strategies include imatinib dose escalation, second-generation TKIs, i.e. dasatinib and nilotinib, allogeneic stem cell transplantation, or clinical trials with an investigational agent. Second-generation TKIs should be particularly mentioned due to their potential to achieve or return and maintain cytogenetic response in approximately 50% of resistant/intolerant CP-CML patients already treated by imatinib [5-7]. Therefore, current medicine offers powerful tools with the potential to improve reachable therapeutic outcomes.

Such remarkable progress deserves relevant methodology quantifying its effect that can be focused either on the efficacy of one particular treatment option or, maybe more importantly, on a patient's health status over the whole follow-up period. Disregarding the treatment sequence and simplifying the patient's status to being in disease remission or not, the course of currently accessible CML treatment can be seen as a series of disease remissions and subsequent relapses. This situation presents a new challenge for attempts to measure therapeutic results, including survival analysis.

Treatment efficacy in patients with leukaemia is usually expressed using either leukaemia-free survival or cumulative incidence. Both approaches are focused on a probability that a pre-defined event will occur in time, e.g. relapse in case of the leukaemia-free survival or disease remission in case of the cumulative incidence. It has to be noted that these estimates focus only on the probability associated with a first occurrence of the event and as such they can be obtained using the well-known product limit estimator [8] which might need to be adjusted for competing risk events [9]. However, since the remission state in CML can currently be achieved repeatedly using several treatment options, patients who relapse after achieving the first disease remission need no longer be considered to have failed the CML treatment. Similarly, the CML in patients who achieve disease remission using the initial imatinib therapy can progress again and these patients need no longer be considered to have remained in CML remission. This implies that the common ways of survival assessment mentioned above are not appropriate for the estimation of the probabilities associated with CML treatment because these measures do not account for the proportion of leukaemia-free patients in subsequent remissions or, conversely, the proportion of patients who have left the remission state.

A quantity adjusting for the subsequent remissions called current leukaemia-free survival (CLFS) was proposed in the literature [10]. Moreover, in 2000, Klein and colleagues [11,12] proposed two new procedures for the CLFS estimation, the first of which is based on a multi-state model, whereas the second is based on the three Kaplan-Meier estimators, and documented its performance on patients transplanted for CML calculating the probability of being in first and second remission after stem cell transplant. The second estimator of Klein et al. is based on an extension of results primarily published by Pepe [13]. The estimation of CLFS in the context of the actual progress in CML therapy has been recently addressed in the work of Al-Kali et al. [14], where a multi-state Markov model was utilized to estimate CLFS. However, the American study focuses mainly on the clinical rather than the methodical aspects of the CLFS estimation.

This work aims to use standard nonparametric statistical methods to meet the following objectives: (1) to estimate the probability that a patient will be in first or in any subsequent CCyR at time *t* after the initiation of imatinib therapy; and (2) to estimate the probability that a patient will stay in first or in any subsequent CCyR at time *t* after achieving the first CCyR on the imatinib therapy. Obviously, the first procedure necessarily takes into account all patients in whom the first-line imatinib therapy has been initiated, whereas the second one counts only with patients who achieved at least one CCyR during the treatment course.

## Methods

### The model

**Figure 1:**
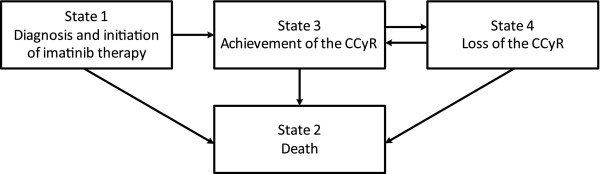
**Block scheme of sequence of possible events in CP-CML patients treated with first-line imatinib**.

The course of currently accessible CML treatment can be described by a model where a patient can be in one of four possible states at a given time point (see Figure 1). Initially, each patient appears in state 1, corresponding to the diagnosis and start of the imatinib therapy. There are two possible transitions from this state: a patient may die without achieving the CCyR (state 2) or achieve the CCyR (state 3). After achieving the CCyR, patients may suffer from disease progression manifested by loss of the CCyR (state 4) or they may die while in remission (state 2). Finally, patients in state 4 may move back to the CCyR (state 3) or they may die (state 2). Obviously, all living patients can move from the CCyR (state 3) to the cytogenetic relapse (state 4) and vice versa repeatedly.

To address the first objective, i.e. to estimate the probability that a randomly selected patient is in his first or any subsequent CCyR at time *t* after the initiation of imatinib therapy, means to quantify the probability of being in state 3 over the interval [0, *t*]. A standard competing risks methodology can be used for this purpose. More specifically, a set of cumulative incidence functions can be used to estimate the probabilities associated with the *i*th achievement of CCyR or the *i*th loss of CCyR, respectively.

Let *r *denote the maximum number of CCyRs achieved in time by any patient. Let *I*_1_(*t*) be the common cumulative incidence function corresponding to the first achievement of CCyR (representing, transition: state 1 → state 3) and *I_i_*(*t*), *i *= 2,..., *r*, be the cumulative incidence functions corresponding to the subsequent achievements of CCyR (representing transitions: state 4 → state 3). Similarly, let $I_{i}^{*}\left(t\right),i=1,\ldots ,r$, be the cumulative incidence functions corresponding to the *i*th loss of CCyR (representing transitions: state 3 → state 4), and $I_{i}^{**}\left(t\right),i=1,\ldots ,r$, be the cumulative incidence functions corresponding to death after the *i*th achievement of CCyR (representing transitions: state 3 → state 2) Then, the probability of state being in first or state any subsequent CCyR at time *t *after the initiation of imatinib therapy, denoted here as the current cumulative incidence of leukaemia-free patients (CCI), can be written using the common cumulative incidence functions:

(1) $$\begin{array}{lll} CCI\left(t\right) & =\overset{r}{\underset{i=1}{\sum }}I_{i}\left(t\right)-\overset{r}{\underset{i=1}{\sum }}I_{i}^{*}\left(t\right)-\overset{r}{\underset{i=1}{\sum }}I_{i}^{**}\left(t\right)\; & \\ & =\overset{r}{\underset{i=1}{\sum }}\left[I_{i}\left(t\right)-I_{i}^{*}\left(t\right)-I_{i}^{**}\left(t\right)\right].\; & \\ & \; & \end{array}$$

Regarding the second objective of this paper, the estimation of the CLFS function, only patients that have ever reached state 3 have to be considered for the estimation process, where the starting time point is the achievement of the first CCyR. Accordingly, we consider only *r *-1 CCyRs that can be achieved in time, indexed with *i *= 2, ..., *r*. Then, let $S_{1}^{*}\left(t\right)$ be the common leukaemia-free survival function, where the event of interest is death in the first CCyR (representing transition: state 3 → state 2) or the first loss of CCyR (representing a first transition from state 3 to state 4), and $S_{i}^{*}\left(t\right),i=2,\ldots ,r$, be the survival functions corresponding to the subsequent losses of CCyR, where the event of interest is death prior to the *i*th loss of CCyR (any transition to state 2 prior to the *i*th transition: state 3 → state 4) or the *i*th loss of CCyR (*i*th transition: state 3 → state 4). Furthermore, let *S_i_*(*t*), *i *= 2,..., *r*, be the survival functions corresponding to the *i*th achievement of CCyR, i.e. patients who die before their *i*th CCyR (any transition to state 2 prior to the *i*th transition: state 4 → state 3) or who have achieved their *i*th CCyR (*i*th transition: state 4 → state 3) are treated as events. Then, the probability of being in first or any subsequent CCyR at time *t *after the achievement of the first CCyR, denoted as CLFS, can be written using the survival functions:

(2) $$CLFS\left(t\right)=S_{1}^{*}\left(t\right)+\overset{r}{\underset{i=2}{\sum }}\left[S_{i}^{*}\left(t\right)-S_{i}\left(t\right)\right].$$

### Nonparametric estimation

To estimate the individual *CCI*(*t*) components, *I_i_*(*t*) and $I_{i}^{*}\left(t\right)$, respectively, we need to associate each patient with a pair of vectors denoted as (*T*_1_,..., *T_r_*, *R*_1_,..., *R_r_*)' and ${\left(T_{1}^{*},\ldots ,T_{r}^{*},R_{1}^{*},\ldots ,R_{r}^{*}\right)}^{\prime }$, respectively, where the former vector represents the CCyR achievements and the latter represents the CCyR losses. Regarding the first of the vectors, *T_i _*is the time to the *i*th event, i.e. the *i*th CCyR or death, or *T_i _*is the censoring time, whereas *R_i _*is the failure cause. When the exact *i*th failure time is not observed, i.e. *T_i _*is censored, then *R_i _*= 0; when the exact failure time is known and the failure cause is the achievement of CCyR, then = *R_i _*= 1; and when the exact failure time is known and the failure cause is death, then *R_i _*= 2. The second vector is organized in the same manner with the exception that the event of interest is the loss of CCyR instead of the CCyR achievement.

Let *λ_i_*(*t*) and $\lambda_{i}^{*}\left(t\right)$ be the cause-specific hazard functions representing the intensity of achieving the *i*th CCyR in time and the intensity of losing the *i*th CCyR in time, respectively. Similarly, let *S_i_*(*t*) and $S_{i}^{*}\left(t\right)$ be the all-cause survival functions considering either the *i*th CCyR or death and the loss of the *i*th CCyR or death, respectively, as the competing causes of failure. Then, assuming independent and identically distributed observations and independent right censoring, the cumulative incidence functions *I_i_*(*t*) and $I_{i}^{*}\left(t\right)$, respectively, can be expressed in the standard way as

(3) $$\begin{array}{lll} I_{i}\left(t\right) & =P\left(T_{i}\leq t,R_{i}=1\right)\; & \\ & =\overset{t}{\underset{0}{\int }}\lambda_{i}\left(u\right)S_{i}\left(u\right)du,\; & \\ & \; & \end{array}$$

(4) $$\begin{array}{lll} I_{i}^{*}\left(t\right) & =P\left(T_{i}^{*}\leq t,R_{i}^{*}=1\right)\; & \\ & =\int_{0}^{t}\lambda_{i}^{*}\left(u\right)S_{i}^{*}\left(u\right)du.\; & \\ & \; & \end{array}$$

The cumulative incidence function corresponding to death after the *i*th achievement of CCyR, $I_{i}^{**}\left(t\right)$, can be expressed in a similar way treating death after the *i*th achievement of CCyR as an event of interest and both death before the *i*th achievement of CCyR and the *i*th loss of CCyR as competing risks.

Regarding the *i*th CCyR achievement and loss, respectively, let *t_i_*_1_,..., *t_ij_*,..., *t_in _*and $t_{i1}^{*},\ldots ,t_{ij}^{*},\ldots ,t_{in}^{*}$ be the observed individual times from the imatinib therapy initiation to the *i*th CCyR achievement and loss, respectively, ranked in ascending order. The *λ_i_*(*t*) and $\lambda_{i}^{*}\left(t\right)$ functions can be then estimated using the Nelson-Aalen estimator [15] which in case of *λ_i_*(*t*) and a particular time point *t_ij _*is of the form ${\hat{\lambda }}_{i}\left(t_{ij}\right)=c_{ij}∕n_{ij}$, where *n_ij _*is the number of patients "at risk" of *i*th CCyR or death at time *t_ij_*, i.e. the number of patients with *T_i _*≥ *t_ij_*, and *c_ij _*is the number of patients achieving the *i*th CCyR at time *t_ij_*, i.e. the number of patients with *T_i _*= *t_ij_*, and *R_i _*= 1. The overall survival functions, *S_i_*(*t*) and $S_{i}^{*}\left(t\right)$, can be estimated using the standard Kaplan-Meier estimator [8] which for *S_i_*(*t*) at *t_ij _*has the form ${Ŝ}_{i}\left(t_{ij}\right)=\prod_{k:t_{ik}\leq t_{ij}}\left(1-d_{ik}∕n_{ik}\right)$, where *d_ik _*is the number of patients achieving the *i*th CCyR or dying at time *t_ik_*, i.e. the number of patients with *T_i _*= *t_ik _*and *R_i _*= 1 or *R_i _*= 2. Incorporating these nonparametric estimates to eq. (3) and (4), *I_i_*(*t*) and $I_{i}^{*}\left(t\right)$ functions, respectively, can be estimated with

(5) $$\begin{array}{c} {\hat{I}}_{i}(t)=\sum_{j:t_{ij}\leq t}{\hat{\lambda }}_{i}(t_{ij}){\hat{S}}_{i}(t_{i(j-1)})\\ =\sum_{j:t_{ij}\leq t}\left[\frac{c_{ij}}{n_{ij}}\prod_{k:t_{ik}<t_{ij}}\left(1-\frac{d_{ik}}{n_{ik}}\right)\right], \end{array}$$

and

(6) $${\hat{I}}_{i}^{*}(t)=\sum_{j:t_{ij}\leq t}{\hat{\lambda }}_{i}^{*}(t_{ij}){\hat{S}}_{i}^{*}(t_{i(j-1)}).$$

Let ${\hat{\lambda }}_{i}^{**}\left(t_{ij}\right)$ be the Nelson-Aalen estimator of the cause-specific hazard function representing the intensity of dying after the *i*th CCyR in time. Then the $I_{i}^{**}\left(t\right)$ function can be estimated with

(7) $${\hat{I}}_{i}^{**}(t)=\sum_{j:t_{ij}\leq t}{\hat{\lambda }}_{i}^{**}(t_{ij}){\hat{S}}_{i}^{*}(t_{i(j-1)}),$$

where ${Ŝ}_{i}^{*}\left(t_{i\left(j-1\right)}\right)$ is the Kaplan-Meier estimate of the all-cause survival function considering either the loss of the *i*th CCyR or death, respectively, as the competing causes of failure. Obviously, the ${Ŝ}_{i}^{*}\left(t_{i\left(j-1\right)}\right)$ estimate in eq. (7) is the same one as in eq. (6).

The model depicted in Figure 1 represents rather a clinical background of the process than the computational aspects. To be able to compare the proposed estimator with that of Klein et al. [11], the model has to be expressed as a progressive multi-state model with each achievement of the CCyR, each loss of the CCyR, and death following either of these possibilities being represented with one state. For example, regarding only two possible disease remissions and two subsequent relapses, the progressive multi-state model has nine states. The first state (state 0) corresponds to CML diagnosis and treatment initiation. Furthermore, a patient may die (state 1) or achieve the first CCyR (state 2). After achieving the first CCyR, a patient may lose the CCyR (state 4) or die (state 3). Once a patient has relapsed, he may die (state 5) or achieve the second CCyR (state 6). Finally, being in the second CCyR, a patient may again relapse (state 8) or die (state 7). Regarding the probability that a randomly selected patient is in his first or second CCyR at time *t* after the initiation of imatinib therapy, our interest is in estimating the probability of being in state 2 or 6.

Let *P*_0*k*_(*t*) be the probability that a patient who was in state 0 at time 0 will be in state *k *at time *t*. Then the cumulative incidences corresponding to the achievements and the losses of CCyR as well as deaths after the *i*th achievement of CCyR can be written as follows:

(8) $$\begin{array}{c} I_{1}\left(t\right)=P_{02}\left(t\right)+P_{03}\left(t\right)+P_{04}\left(t\right)+P_{05}\left(t\right)\\ \;\;\;+P_{06}\left(t\right)+P_{07}\left(t\right)+P_{08}\left(t\right), \end{array}$$

(9) $$\begin{array}{c} I_{1}^{*}\left(t\right)=P_{04}\left(t\right)+P_{05}\left(t\right)+P_{06}\left(t\right)+P_{07}\left(t\right)\\ \;\;\;+P_{08}\left(t\right), \end{array}$$

(10) $$I_{1}^{**}\left(t\right)=P_{03}\left(t\right),$$

(11) $$I_{2}\left(t\right)=P_{06}\left(t\right)+P_{07}\left(t\right)+P_{08}\left(t\right),$$

(12) $$I_{2}^{*}\left(t\right)=P_{08}\left(t\right),$$

(13) $$I_{2}^{**}\left(t\right)=P_{07}\left(t\right).$$

Adding and subtracting terms given by eq. (8) - (13) according to eq. (1) we get *CCI*(*t*) = *P*_02_(*t*) + *P*_06_(*t*), which is exactly the same expression as we would have obtained considering the estimation of the probability of being in state 2 or 6 with the Markov multi-state model of Klein et al. [11]. The difference between these two estimators is in a way how the probabilities of interest are estimated. Regarding the Markov model, the transition probabilities are estimated directly using the estimates ${\hat{P}}_{02}\left(t\right)$ and ${\hat{P}}_{06}\left(t\right)$ based on the estimated transition probability matrix, whereas the expression proposed in this paper suggests estimating the probabilities of interest in an indirect manner using the Aalen-Johansen estimates of the cumulative incidences. Comparison of the Markov model and the current cumulative incidence estimates of the probability that a patient will be in any CCyR at time *t* after the initiation of imatinib therapy using the Czech CML data is provided in the Results section.

To estimate *S_i_*(*t*) and $S_{i}^{*}\left(t\right)$ in eq. (2), respectively, the vectors characterizing each patient need to be rewritten with respect to the fact that the starting time point is the achievement of the first CCyR. In other words, we need to adjust all event times for the time *T*_1 _representing the time to the first CCyR. The vectors can be rewritten as (*T*_2 _- *T*_1_,..., *T_r _*- *T*_1_, *R*_2_,..., *R_r_*)' and ${\left(T_{1}^{*}-T_{1},\ldots ,T_{r}^{*}-T_{1},R_{1}^{*},\ldots ,R_{r}^{*}\right)}^{\prime }$. Obviously, only patients with *R*_1 _= 1 are used for the estimation of *S_i_*(*t*) and $S_{i}^{*}\left(t\right)$. Both functions can be easily estimated using the above mentioned Kaplan-Meier estimator regarding *i*th achievement of CCyR or death before the *i*th CCyR and *i*th loss of CCyR or death before the *i*th loss of CCyR, respectively, as the events of interest. Let ${Ŝ}_{i}\left(t\right)$ and ${Ŝ}_{i}^{*}\left(t\right)$ be the Kaplan-Meier estimators of *S_i_*(*t*) and $S_{i}^{*}\left(t\right)$, respectively. Then, the *CLFS*(*t*) estimator is given by

(14) $$\hat{CLFS}\left(t\right)={Ŝ}_{1}^{*}\left(t\right)+\overset{r}{\underset{i=2}{\sum }}\left[{Ŝ}_{i}^{*}\left(t\right)-{Ŝ}_{i}\left(t\right)\right].$$

It should be noted that eq. (14) is principally the same as the nonparametric estimator proposed in Klein et al. [11]. However, their model considered only two remission phases, i.e. they considered *r *= 2. In the context of the current CML therapy, we anticipate multiple disease remissions and relapses over time, which means that *r *will be dependent mainly on the follow-up of the particular group of patients.

So far, only point estimates were considered. However, confidence intervals (CI) are also necessary to fully cover the variability of the point estimates. Standard error estimator can be derived using theoretical results published by Pepe [13] and Lin [16]; however, it is neither mathematically nor computationally simple. Bootstrapping represents a feasible alternative for the estimation of the 100(1 - *α*)% confidence bands. Given the observed data, either Efron's bootstrap procedure I or II [17] can be used to resample from randomly right-censored observations. The Efron's procedure II simply draws random samples with replacement from the observed vectors of survival times and corresponding censoring indicators. On the other hand, the procedure I considers separate resampling with replacement from the empirical distribution of survival times and the empirical distribution of censoring times. The empirical distributions are estimated from data by the Kaplan-Meier estimator, and the bootstrap sample observations are then obtained taking minima and corresponding indicators. The confidence bands can then be estimated using the percentile method, where, for example, the 2.5 and the 97.5 percentiles of the estimated bootstrap distribution can be used as the limits of the 95% confidence interval.

## Results

To demonstrate the usability of the proposed statistical estimates, we applied them to representative records on all consecutive CP-CML patients treated by first-line imatinib in two Czech haematological centres in Prague and Brno [18]. In total, 152 consecutively recorded CP-CML patients received the first-line imatinib between July 2003 and July 2009; all records were registered in the Czech database called INFINITY (tyrosine kinase Inhibitors iN FIrst aNd followIng CML Treatment). In all patients, the treatment response was evaluated according to the European Leukaemia Net (ELN) recommendations [19]. The median age of the patients recorded in the examined data set (N = 152) was 55 years with range 20 - 77 years, 69 (45.4%) were male, and median follow-up from the start of imatinib therapy was 35.9 months with range 10.8 - 74.4 months. Regarding treatment response, 128 patients achieved at least first CCyR and 2 patients died without achieving any CCyR. Of the 128 first remission patients, 31 lost the first CCyR and 3 patients died. Furthermore, 18 of the 31 relapsed patients achieved a second CCyR (11 with imatinib, 6 with dasatinib, and 1 with nilotinib) and 4 patients died in the first relapse. Of the 18 second remission patients, two lost the second CCyR and the both patients achieved the third CCyR (1 with dasatinib, and 1 with nilotinib).

**Figure 2:**
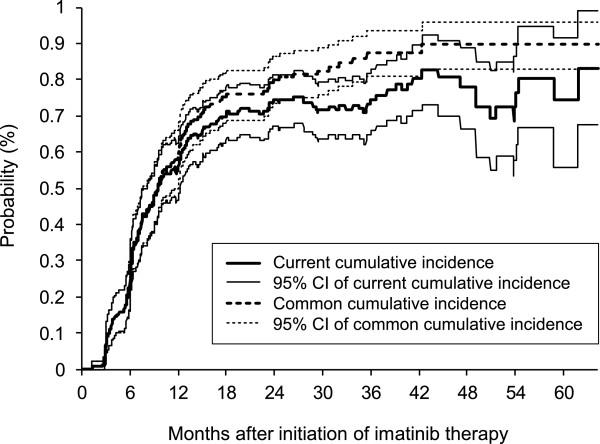
**Estimates of current and common cumulative incidence functions with 95% point-wise bootstrap confidence intervals (N = 152)**.

Figure 2 shows the estimates of the common cumulative incidence function, ${\hat{I}}_{1}(t)$, and the current cumulative incidence function, $\hat{CC}I\left(t\right)$, as well as the 95% point-wise bootstrap confidence intervals. Point estimates and confidence intervals are further summarized in Table 1; the estimates are given for meaningful times when responses are assessed according to ELN criteria [19]. As expected, the common cumulative incidence curve overestimates the probability of being alive and in remission after the initiation of the imatinib therapy because it doesn't take into account the fact that some patients can lose and achieve their remission repeatedly. The estimated proportion of patients who have left the first CCyR state after its achievement reached the maximum of 12.8% at 3 years after the start of the therapy. Moreover, we can see that the 95% point-wise bootstrap confidence interval of the current cumulative incidence curve is wider than the corresponding confidence interval of the common cumulative incidence curve as time increases. However, this can be expected as several processes are combined in the current cumulative incidence implying that the heterogeneity of the cumulative incidences corresponding to the subsequent achievements and losses of CCyR adds on to the heterogeneity of the common cumulative incidence.

**Table 1:** Comparison of current and common cumulative incidence function estimates in time (N = 152).

Time	**Common cumulative incidence function estimate: **${\hat{I}}_{1}\left(t\right)$		**Current cumulative incidence function estimate: **$\hat{CC}I\left(t\right)$		Difference
	(%)	95% CI	(%)	95% CI	(%)
3 months	7.2	3.3 - 11.8	7.2	3.3 - 11.8	0.0
6 months	26.3	19.7 - 33.6	25.7	19.1 - 32.2	0.7
12 months	58.6	50.7 - 66.4	55.3	47.4 - 63.1	3.3
18 months	75.9	68.8 - 82.5	71.3	63.9 - 78.5	4.6
24 months	79.8	73.2 - 86.3	74.4	67.0 - 81.4	5.4
36 months	87.3	80.9 - 93.3	74.5	66.0 - 82.8	12.8
48 months	89.6	82.8 - 96.0	77.9	66.2 - 88.9	11.6

**Figure 3:**
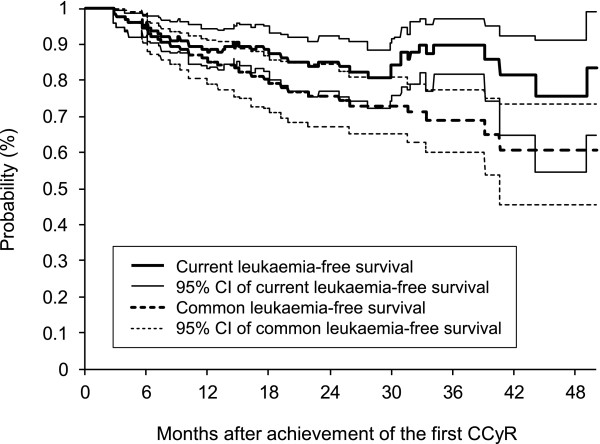
**Estimates of current and common leukaemia-free survival functions with 95% point-wise bootstrap confidence intervals (N = 128)**.

The estimates of the common leukaemia-free survival function, ${Ŝ}_{1}^{*}\left(t\right)$, and of the current leukaemia-free survival function, $\hat{CLFS}\left(t\right)$, are compared in Figure 3 together with their 95% point-wise bootstrap confidence intervals. The estimates are further numerically summarized in Table 2. As in the case of the cumulative incidence, we can see the discrepancy between the current and common leukaemia-free survival curves reflecting the probability of being alive in second and subsequent remissions that can be achieved using currently available CML therapy. Regarding the currently available follow-up period, this discrepancy also takes its maximum value at 3 years after the first achievement of the CCyR, where the difference between the two curves reaches 20.8%.

**Table 2:** Comparison of current and common leukaemia-free survival function estimates in time (N = 128).

Time	**Common leukaemia-free survival function estimate: **${\hat{S}}_{1}^{*}\left(t\right)$		**Current leukaemia-free survival function estimate: **$\hat{CLFS}\left(t\right)$		Difference
	(%)	95% CI	(%)	95% CI	(%)
3 months	98.4	96.0 - 100.0	98.4	96.0 - 100.0	0.0
6 months	94.5	90.5 - 97.6	95.3	92.0 - 98.1	-0.8
12 months	85.2	78.9 - 91.0	87.6	83.1 - 94.2	-2.5
18 months	79.1	71.1 - 85.6	87.4	80.3 - 93.3	-8.3
24 months	75.6	67.3 - 84.6	85.1	77.0 - 92.4	-9.5
36 months	68.8	60.0 - 77.2	89.7	81.7 - 97.2	-20.8
48 months	60.7	45.4 - 73.3	75.5	54.6 - 91.2	-14.8

**Figure 4:**
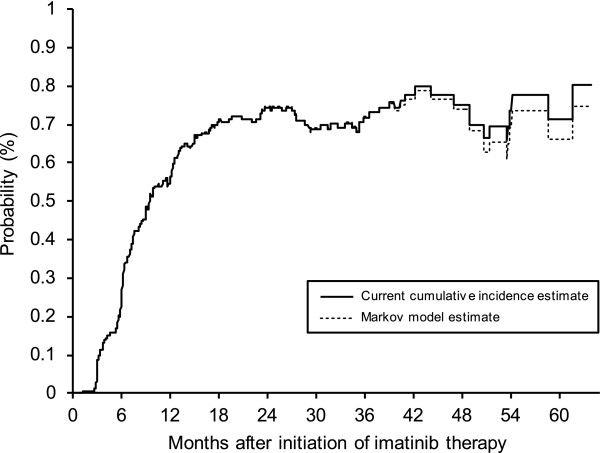
**Comparison of the current cumulative incidence estimates based on the Markov multi-state model and the proposed method using the Czech CML data**.

Figure 4 shows the estimated current cumulative incidence curves calculated by means of the method of Klein et al. and the proposed estimator on the Czech CML data. It can be seen that both estimates are almost identical up to 36 months of follow-up and are very similar onwards. However, the estimates become more divergent when less than 10 patients remain under follow-up. For example, at 3 years, the difference between the two estimates is 0.1%, whereas at 5 years, the difference between the estimates is 5.3%.

Moreover, a simulation study was employed to assess the performance of our estimators and the Markov model estimator of Klein et al. [11]. We considered the four-state model introduced in Figure 1 with times to the *i*th achievement or loss of CCyR generated using a semi-Markov process with Weibull distribution of the transition times [20,21]. Shape and scale parameters of the Weibull distribution were estimated individually for each transition using the Czech CML data. Times to death were also generated from the Weibull distribution with data-driven parameters. Any transitions that occurred after the simulated time of death were not used in the estimation. In total, 10,000 independent empirical curves calculated using exact transition times without censoring were used to approximate the true curve characterising the underlying process. The performance of all estimators was assessed using censored data (uniform distribution regarding the follow-up period from 0 to 10 years was used to generate censoring times). In total, 1,000 independent observations from the process with sample sizes 100 and 1,000 were used to estimate the cumulative incidence curve. Another 1,000 independent observations were considered for each sample size in order to estimate the leukaemia-free survival curve.

The results are given in Tables 3 and 4, summarizing for all estimators and both sample sizes median absolute deviations (MAD) between the estimated and the true curves, as well as proportions of the 95% confidence intervals that contained the approximated true curve (denoted as coverage of the 95% CI). According to the MAD, both tables show good performance of the evaluated estimators in the case of the larger sample size. Even after ten years from diagnosis, half of the current cumulative incidence estimates and the corresponding Markov model estimates based on the larger sample size are closer to the true value than 2.0% and 1.9%, respectively (Table 3). Regarding the current leukaemia-free survival estimator and the corresponding Markov model estimator, half of the estimates based on the larger sample size are closer to the true value than 2.2% and 2.1%, respectively (Table 4). As for the smaller sample size, the variability of the estimates is higher; however, both current cumulative incidence and current leukaemia-free survival estimators have comparable performance to the Markov model estimator. More importantly, Tables 3 and 4 also show that the new estimators and the Markov model estimator are fully comparable regarding the coverage of their 95% point-wise confidence intervals; the only exceptions are the estimates based on the smaller sample size at nine and ten years after diagnosis where the Markov model confidence interval coverage reached only about 90%. The nominal coverage of the 95% point-wise bootstrap confidence intervals is similar for both sample sizes.

**Table 3:** Simulation results for the current cumulative incidence estimator and the Markov model estimator of Klein et al.

	Sample size 100				Sample size 1,000			
	CCI estimator		Markov model estimator		CCI estimator		Markov model estimator	
Time								
	MAD* (%)	Coverage of the 95% CI** (%)	MAD* (%)	Coverage of the 95% CI**(%)	MAD* (%)	Coverage of the 95% CI** (%)	MAD* (%)	Coverage of the 95% CI** (%)
1 year	3.7	93.8	3.7	92.8	1.1	95.4	1.1	95.2
2 years	3.2	94.7	3.2	94.4	1.0	94.4	1.0	94.5
3 years	3.1	94.3	3.1	94.2	1.0	95.2	1.0	95.2
4 years	3.2	95.0	3.2	94.3	1.0	95.4	1.0	95.1
5 years	3.6	95.4	3.6	94.7	1.0	93.7	1.0	94.7
6 years	3.6	95.7	3.6	94.5	1.2	92.9	1.2	94.2
7 years	4.2	94.6	4.0	94.0	1.2	95.9	1.2	96.2
8 years	4.8	95.5	4.7	93.4	1.5	94.9	1.4	94.8
9 years	5.7	92.9	5.7	90.4	1.6	95.3	1.6	95.6
10 years	6.4	92.3	6.4	87.3	2.0	94.1	1.9	94.9

* Median absolute deviation between the estimated and true curve.

** The proportion of 95% point-wise confidence intervals that contained the approximated true curve.

**Table 4:** Simulation results for the current leukaemia-free survival estimator and the Markov model estimator of Klein et al.

	Sample size 100				Sample size 1,000			
	CLFS estimator		Markov model estimator		CLFS estimator		Markov model estimator	
Time								
	MAD* (%)	Coverage of the 95% CI** (%)	MAD* (%)	Coverage of the 95% CI** (%)	MAD* (%)	Coverage of the 95% CI** (%)	MAD* (%)	Coverage of the 95% CI** (%)
1 year	2.3	92.7	2.3	92.7	0.7	93.5	0.7	94.2
2 years	2.7	94.5	2.7	94.3	0.8	94.3	0.9	94.6
3 years	3.0	94.3	3.0	93.2	1.0	95.2	1.0	95.2
4 years	3.4	94.2	3.4	94.1	1.0	94.9	1.0	95.9
5 years	3.5	93.6	3.8	93.3	1.2	93.1	1.2	94.7
6 years	4.1	93.9	4.0	93.2	1.2	95.2	1.3	95.2
7 years	4.2	93.6	4.1	92.5	1.5	95.0	1.4	93.9
8 years	5.0	95.2	4.7	93.2	1.6	94.0	1.5	93.8
9 years	5.8	94.3	5.6	90.1	1.9	93.3	1.8	94.0
10 years	6.7	94.2	6.3	88.1	2.2	95.1	2.1	92.3

* Median absolute deviation between the estimated and true curve.

** The proportion of 95% point-wise confidence intervals that contained the approximated true curve.

## Discussion

Recent progress in CML treatment opens a challenging field for the application and training of statistical methods. The "perspectives" rest especially in various treatment options and multiple measures of treatment efficacy that have been introduced recently with TKI therapy. However, there is no paper addressing the issue of multiple disease remissions in time besides the work of Klein et al. [11] that has been further utilized in the article of Al-Kali et al. [14].

We have presented two quantities for estimating two principal characteristics of the current CML treatment: (1) the probability of being alive and in CCyR in time after CML therapy initiation; and (2) the probability that a patient is alive and in the first or any subsequent CCyR in time after achieving the first CCyR on the CML treatment. Both quantities result from a clinical model proposed to enable CML patients to move repeatedly between disease status and disease-free status, and can be expressed using the well-known statistics, namely the cumulative incidence functions and the survival functions, which are familiar and easily interpretable for both data analysts and clinicians. Moreover, the probabilities of interest can be estimated using standard nonparametric statistical methods, which are commonly used in survival analysis [8], competing risks analysis, and estimation of the stage occupation probabilities in multi-state models [20]. More specifically, the estimator regarding the probability of being alive and in CCyR in time after CML therapy initiation is expressed as a linear combination of the Aalen-Johansen estimators, whereas the procedure regarding the probability that a patient is alive and in the first or any subsequent CCyR in time after achieving the first CCyR is expressed as a linear combination of the Kaplan-Meier estimators. The later method can thus be seen as a slight adjustment of the CLFS estimation technique introduced previously by Klein et al. [11,12].

The two methods represent nonparametric and easy-to-use estimators of the above mentioned probabilities with death from any reason being considered as a competing risk. The main advantage of the proposed approaches is the computational simplicity of the point estimation where standard software tools that are widely accessible for data analysts as well as for clinicians can be used (all computations presented in this paper were obtained using the R statistical software [22]). On the other hand, the calculation of the confidence intervals is not so easy. A standard error estimator can be derived using the asymptotic theory published by Pepe [13] and results of Lin [16]; however, it is neither mathematically nor computationally simple. Moreover, its precision may be questionable due to underestimation bias with respect to the true variances as published in [13]. With respect to these facts, an alternative bootstrapping procedure with 10,000 bootstrap samples was adopted to estimate the 95% confidence intervals in this paper.

Another approach to estimate the probabilities of interest is to use a multi-state Markov model [11,14]. This model also enables the estimation of the probability that a patient is in any one of the possible states, as well as it allows us to quantify the standard error of such estimates. Moreover, covariate effects can be incorporated into the multi-state Markov model using a regression model for each of the transition rates [23]. The reason why we regard the Markov model potentially inappropriate for the problem presented in this paper is the necessary assumption of the Markovian nature of the transition probabilities. This assumption is dubious, as we suppose that there can be a difference in the length of the remission period in two patients achieving their first remission in two different times (e.g. after 3 and 18 months from the start of imatinib therapy) as we can anticipate different characteristics of the disease and thus also a different disease behaviour [4,24]. However, according to a proportional hazards model which included the time spent in a particular state as a covariate, we found no evidence of a departure from the Markov assumption in our data. This result may suggest validity of the Markov assumption in such a data but it should be noted that the considered data set is relatively small (N = 152) and the number of possible disease remissions that can be achieved in time is limited. On the other hand, regarding the good performance of the Markov model estimator of Klein and colleagues on the simulated data coming from a semi-Markov process we can suppose that this estimator performs relatively well also for non-Markov structures. In general, we can conclude that alternative methods such as semi-Markov models or fully nonparametric methods can be used for the estimation when the Markov assumption does not hold [25].

Even if the explicit form of the Aalen-Johansen estimator of the cumulative incidence in competing risks assumes the Markovian nature of the transition probabilities, the usability of this estimator is not restricted only on Markov models, because Datta and Satten [21] showed that the Aalen-Johansen estimator is consistent also for non-Markov models provided that the censoring times are independent of the states occupied by the individuals and the transition times between the states. On the other hand, this implies a necessary assumption that the censoring is independent of the states occupied and the transition times between the states, which one should be aware of prior to data analysis. When this assumption is violated, the estimator may be modified using the so-called inverse probability of censoring weighting that was originally proposed by Datta and Satten [26].

An issue that should also be considered when regarding the proposed methods is the clinical relevance of the underlying model. The proposed model is compatible with the current CML therapy in two essential points. First, it respects the course of currently accessible CML treatment as a series of disease remissions and subsequent relapses, i.e. it can adequately reflect the dynamics in CML patient health status. Second, the model focuses on the whole treatment process rather than on the individual treatment option represented by single drugs. From this point of view, the model as well as the proposed methodology is also applicable in the future, regardless of what the progress in the CML treatment will be and how many treatment options will be available, respectively.

Another important issue is the practical value of the current cumulative incidence of the leukaemia-free patients and the current leukaemia-free survival as survival measures. As already mentioned, both quantities outperform the commonly used measures like leukaemia-free survival and cumulative incidence of the disease-free patients when describing the CML patient health status. Comparing the interpretation value of these two quantities to the overall survival (OS), both advantages and disadvantages can be seen. Obviously, the OS remains the gold standard for efficacy evaluation as it can be with no doubt assessed easily and accurately, and, what is even more important, statistically significant improvement in OS also implies almost in every case a result of practical significance [27]. This is in contrast with the assessment of the treatment response where several biases can occur, e.g. measurement bias or evaluation time bias [28]. On the other hand, the OS of CML patients has improved dramatically due to recent developments in CML therapy, and its reliable evaluation now requires a sufficiently long follow-up. Furthermore, high OS rates are also associated with an increasing number of patients needed for the proper estimation, which is often unfeasible for epidemiological reasons. These practical problems justify the use of the current cumulative incidence of the disease-free patients and the current leukaemia-free survival as the primary measures for assessment of the CML treatment.

A future challenge is associated with the methods of statistical inference for the nonparametric estimation procedures presented here; especially a test for comparison of the two estimated curves and a confidence interval for the difference of two estimated curves would be of interest. Results of Liu et al. [29] and Lin [16] may be useful for this research.

## Conclusions

Two quantities for the evaluation of the efficacy of current CML therapy that may be estimated with standard nonparametric methods have been proposed in this paper. Both quantities reliably illustrate a patient's disease status in time because they account for the proportion of patients who have left the first disease remission, as well as for the proportion of leukaemia-free patients being in second and subsequent disease remissions. Their usability was demonstrated in the data of 152 consecutive CP-CML patients treated in the two largest haematological centres in the Czech Republic. Moreover, the model is also applicable in the future, regardless of what the progress in the CML treatment will be and how many treatment options will be available, respectively. The overall novelty of this paper can be seen mainly in the clinical model and the easiness of the way how the well-known statistical estimators can be combined to estimate the probabilities of interest.

## Competing interests

The authors declare that they have no competing interests.

## Authors' contributions

TP and EJ proposed the model, carried out the computations and drafted the manuscript. ZP and JMu participated in the computations and data management. DZ, ZR, HK, PC and MT contributed to study design, and collected the data. JMa and LD proposed the idea for the study, and participated in the manuscript. All authors read and approved the final manuscript.

## Pre-publication history

The pre-publication history for this paper can be accessed here:

http://www.biomedcentral.com/1471-2288/11/140/prepub
